# Establishment of a Parvovirus B19 NS1-Expressing Recombinant Adenoviral Vector for Killing Megakaryocytic Leukemia Cells

**DOI:** 10.3390/v11090820

**Published:** 2019-09-04

**Authors:** Peng Xu, Xiaomei Wang, Yi Li, Jianming Qiu

**Affiliations:** 1 Hubei Engineering Research Center of Viral Vector, Wuhan University of Bioengineering, Wuhan 430415, China; 2 Department of Microbiology, Molecular Genetics and Immunology, University of Kansas Medical Center, Kansas City, KS 66160, USA

**Keywords:** parvovirus B19, adenoviral vector, cell cycle arrest, apoptosis, anti-cancer

## Abstract

Adenoviral viral vectors have been widely used for gene-based therapeutics, but commonly used serotype 5 shows poor transduction efficiency into hematopoietic cells. In this study, we aimed to generate a recombinant adenovirus serotype 5 (rAd5) vector that has a high efficiency in gene transfer to megakaryocytic leukemic cells with anticancer potential. We first modified the rAd5 backbone vector with a chimeric fiber gene of *Ad5* and *Ad11p* (rAd5F11p) to increase the gene delivery efficiency. Then, the nonstructural protein NS1 of human parvovirus B19 (B19V), which induces cell cycle arrest at the G2/M phase and apoptosis, was cloned into the adenoviral shuttle vector. As the expression of parvoviral NS1 protein inhibited Ad replication and production, we engineered the cytomegalovirus (CMV) promoter, which governs NS1 expression, with two tetracycline operator elements (TetO_2_). Transfection of the rAd5F11p proviral vectors in Tet repressor-expressing T-REx-293 cells produced rAd in a large quantity. We further evaluated this chimeric rAd5F11p vector in gene delivery in human leukemic cells, UT7/Epo-S1. Strikingly, the novel rAd5F11p-B19NS1-GFP vector, exhibited a transduction efficiency much higher than the original vector, rAd5-B19NS1-GFP, in UT7/Epo-S1 cells, in particular, when they were transduced at a relatively low multiplicity of infection (100 viral genome copies/cell). After the transduction of rAd5F11p-B19NS1-GFP, over 90% of the UT7/Epo-S1 cells were arrested at the G2/M phase, and approximately 40%–50% of the cells were undergoing apoptosis, suggesting the novel rAd5F11P-B19NS1-GFP vector holds a promise in therapeutic potentials of megakaryocytic leukemia.

## 1. Introduction

Adenovirus (Ad) is a nonenveloped icosahedral virus and has a double-stranded DNA genome of 30 to 38 kbp. Ad has been studied intensively for over 50 years in models of virus–cell interactions, cellular processing, and latterly as a gene delivery vector. To date, over 60 serotypes of human Ad have been described, most of which infect the respiratory or gastrointestinal tracts and the eye [1]. Recombinant Ad (rAd) is one of the most popular gene delivery vectors and has been used constantly for phase I to III clinical trials in the development of vaccines and therapeutic gene transfer in the last 20 years [2]. The reasons for why rAd is widely used for gene vectors are that Ad has a wide range of tropism to cells and tissues, and infections are not associated with serious pathogenicity in general [3]. rAd vectors have been proved to efficiently deliver transgenes to the nucleus of a wide range of cell types and then mediate a high level of expression of the transgenes. Moreover, rAd vectors transduce both proliferating and differentiated cells [4]. After infection, rAd vectors remain episomal and do not integrate into the host cell genome, which minimizes the risk of insertional mutagenesis. Furthermore, rAd vectors have a remarkable DNA packaging capacity, offering possibilities for genetic manipulations. Last, rAd vectors are easy to produce, requiring very limited ‘hands on’ time from shuttle/backbone plasmid cotransfection to the isolation of virus particles. 

However, the commonly used Adenovirus serotype 5 (*Ad5*) has a poor transduction efficiency in hematopoietic cells, mainly because of the low expression of *Ad5* receptors on the cells [5]. For Ad entering the cells, the key step is the interaction between viral fiber knob and its cellular receptor. So, a method for changing the knob domain or even the entire fiber gene of *Ad5* is used to improve the gene delivery efficiency to target cells. Different Ad serotypes exhibit different tissue tropism because of the different fiber proteins. *Ad11p*, which belongs to the human species B adenoviruses, was reported to have the ability to infect human CD34^+^ hematopoietic cells [6]. Therefore, the chimeric Ad vector containing a chimeric fiber protein between *Ad5* and *Ad11p* fibers may have higher transduction efficiency to hematopoietic cells than the *Ad5* vector.

Parvovirus B19 (B19V) belongs to the *Erythroparvovirus* genus within the *Parvoviridae* family [7]. B19V has a high tropism for the human erythroid progenitor cells (EPCs) from the bone marrow and fetal liver [8]. B19V has a linear single stranded DNA (ssDNA) genome of approximately 5.6 kb, which has identical inverted terminal repeats (ITRs) of 383 nucleotides at both ends [9]. The double-stranded replicative-form (RF) DNA of the B19V genome encodes a large nonstructural protein (NS1). B19V NS1, 671 amino acids (aa) in length, has a molecular weight of approximately 78 kDa. NS1 predominantly localizes in the nucleus of infected cells, as it contains nuclear localization signals at amino acid residues 177 to 180 (KKPR) and 316 to 321 (KKCGKK) [10]. B19V NS1 not only plays important roles in viral DNA replication and transcription activation [11], but also executes a cytotoxic effect on human erythroid cells. B19V-infected cells have ultrastructural features associated with apoptosis, and the NS1 has been identified as the apoptosis-inducer [12,13]. Besides, we have previously found that B19V NS1 induces cell cycle arrest at the G2/M phase [14]. 

Leukemias are a group of life-threatening malignant disorders of the blood and bone marrow. Despite significant progress achieved in the past decade in the chemotherapy-based and targeted treatments of several leukemia subsets, relapse remains common after an initial response, indicating the resistance of leukemia cells to current therapies. 

In this study, we modified the *Ad5* fiber gene in rAd5 with the *Ad11P* fiber gene to increase the transduction efficiency into UT7/Epo-S1 cell line, which was generated by Kazuo Sugamura and is susceptible to B19V infection [15]. UT7/Epo-S1 is a subline derived from UT7/Epo, which is an erythropoietin (Epo)-dependent cell line, and originating from UT7, a megakaryocytic leukemia cell line [16]. We demonstrated that the transduction of the chimeric rAd that expresses B19V NS1-induced cell cycle arrest and apoptosis. Thus, our novel rAd5F11P-B19NS1 vector, which can be subjected to further genetic manipulations, holds promise for gene-based therapeutic medicine of leukemia treatment.

## 2. Material and Methods

### 2.1. Cell Lines

HEK293 cells (CRL-1573) were purchased from the American Type Culture Collection (ATCC) (Manassas, VA, USA) and were cultured in Dulbecco’s modified Eagle’s medium (DMEM; GE Healthcare Biosciences, Piscataway, NJ, USA) with 10% fetal calf serum (Sigma-Aldrich, St. Louis, MO, USA). 

T-REx-293 cells were purchased from Invitrogen (Carlsbad, CA, USA) and were cultured in DMEM plus 10% fetal calf serum and 10 μg/mL blasticidin. 

UT7/Epo-S1 cells were obtained from Dr. Kevin Brown at the Hematology Branch, NHLBI, NIH, with permission from Dr. Kazuo Sugamura at Tohoku University, Japan, and were cultured under normoxic conditions in DMEM containing 10% fetal calf serum (FCS) and 2 U/mL erythropoietin (Amgen, Thousand Oaks, CA, USA).

### 2.2. Plasmids

The DNA sequences coding B19V NS1 were codon-optimized and purchased at GenScript USA Inc. (Piscataway, NJ, USA) as previously described [17]. 

The pacAd5 CMV-GFP transfer plasmid was made by digesting the pacAd5 U6-GFP (purchased from Cell Biolabs (San Diego, CA, USA)) with XhoI to remove the mU6 promoter and multiple cloning site (MCS). Then the CMV promoter, MCS, and a bGH poly(A) signal were inserted. 

The CMV promoter of the pacAd5 CMV-GFP vector was modified by inserting two repeated tetracycline operator elements (5′-TCC CTA TCA GTG ATA GAG ATC TCC CTA TCA GTG ATA GAG A-3′) at 9 bases behind the TATA box, resulting in pacAd5 CMVTetO_2_-GFP. C-terminally Strep-tagged optimized (opt) B19V NS1 was inserted into pacAd5 CMVTetO_2_-GFP to generate the transfer plasmid, pacAd5 CMVTetO_2_-B19NS1-GFP.

pacAd5 9.2-100 was purchased from Cell Biolabs, Inc. (San Diego, CA, USA) The fiber gene in the pacAd5 9.2-100 was replaced with a chimeric fiber gene encoding the *Ad5* fiber tail domain and *Ad11p* fiber shaft and knob domains described as before [18]. Firstly, pacAd5 9.2-100 was digested into two fragments by EcoRI. The large fragment of 26,438 bp was saved for use, the small fragment of 8509 bp which contains *Ad5* fiber gene was ligated to EcoRI-digested pGEX-3Z to generate pGEX-3Z-Ad5F. Secondly, chimeric fiber gene Ad5F11P was synthesized using an overlapping PCR strategy, with AgeI or Alf II site at each end. Thirdly, AgeI/Alf II fragment that encodes *Ad5* fiber gene from pGEX-3Z-Ad5F was replaced with the synthesized Ad5F11P digested by the same enzymes to form a new plasmid, pGEX-3Z-Ad5F11P. Finally, pGEX-3Z-Ad5F11P was digested with EcoRI, and ligated with the large fragment of the EcoRI-digested pacAd5 9.2-100 to generate pacAd5F11P 9.2-100.

All plasmids were sequenced to confirm their constructions at MCLAB (South San Francisco, CA, USA).

### 2.3. Recombinant Adenoviral (rAd) Vector Construction and Purification

rAd was made following the instructions provided by Cell Biolabs (San Diego, CA, USA). Briefly, linearized shuttle vector pacAd5 CMVTetO_2_-GFP or pacAd5 CMVTetO_2_-B19NS1-GFP were transfected into T-REx-293 cells with linearized Ad genome backbone, pacAd5 9.2-100 or pacAd5F11P 9.2-100. After one week, when plaques were formed, cells were collected and resuspended in phosphate buffered saline (PBS), pH 7.4, and lysed by three cycles of freezing and thawing. Crude viral lysates were collected for virus propagation. The final viral lysates were spun at 10,000 rpm for 30 min, and then the supernatant was collected and dissolved in CsCl at a density of 1.36 g/mL for centrifugation in a Sorvall TH641 rotor at 36,000 rpm for 36 h at 20 °C Fractions of 500 μL were collected using a Piston Gradient Fractionator (BioComp, Fredericton, NB, Canada).

### 2.4. Q-PCR

The primers and FAM (6-carboxyfluorescein)-labeled probe used for quantification of rAd DNA were designed to target the *GFP* gene: Forward primer (5′-CTG CTG CCC GAC AAC CA-3′), Reverse primer (5′-TGT GAT CGC GCT TCT CGT T-3′), and the probe (5′-FAM-TAC CTG AGC ACC CAG TCC GCC CT-BHQ1-3′). Quantitative PCR was performed as described previously [19] on a 7500 Fast real-time PCR system (Applied Biosystems, Foster City, CA, USA). 

### 2.5. Fluorescence Images 

GFP fluorescence images were taken at a magnification of 10 × (objective lens), with a Nikon Eclipse Ti-S inverted microscope (Nikon, Tokyo, Japan).

### 2.6. Flow Cytometry Analysis

(i) Cell cycle analysis: Cell cycle analysis was performed using the 4′,6-diamidino-2-phenylindole (DAPI) staining described as before [20]. Briefly, rAd-transduced UT7/Epo-S1 cells were washed with PBS, fixed by 1% Paraformaldehyde (PFA), then permeabilized with 0.4% tween-20 and stained with DAPI at a concentration of 1 μg/mL for 30 min in dark. Samples were analyzed by flow cytometry within 1 h.
(ii) Fluorescent-Labeled Inhibitors of Caspases (FLICA): FLICA Caspase-9 assay kit was purchased from ImmunoChemistry Technologies (Bloomington, MN, USA) and the assay was performed following the manufacturer’s protocol. Briefly, 290 µL of rAd-transduced UT7/Epo-S1 cells was incubated with 10 µL of the working solution of the reagent and then incubated for approximately 1 h, and then analyzed with a flow cytometer.
(iii) Apoptosis analysis: FITC-conjugated Annexin V and Propidium Iodide (PI) double staining was performed following the manufacturer’s protocol. Briefly, rAd-transduced UT7/Epo-S1 cells were washed twice with cold PBS and then resuspended in 1 × Binding Buffer at a concentration of 1 × 10^6^ cells/mL. A volume of 100 µL of the solution (1 × 10^5^ cells) was transferred to a 1.5 mL tube, with 5 µL of FITC Annexin V and 5 µL of PI. After gentle vertexing, the cells were incubated for 15 min at RT (25 °C) in dark, with 400 µL of 1 × Binding Buffer, and were analyzed by flow cytometry within 1 h.

All processed samples were analyzed on a three-laser flow cytometer (LSR II; BD Biosciences (San Jose, CA, USA)) at the Flow Cytometry Core at the University of Kansas Medical Center. All flow cytometry data were analyzed using FACS DIVA software (BD Biosciences).

### 2.7. Western Blot

Cells were transfected or transduced as indicated in each figure. The cells were harvested and lysed 2 days post-transfection/transduction. Western blotting was performed to analyze the lysates as previously described [21], using anti-strep and β-actin antibodies. 

## 3. Results

### 3.1. Modification of the rAd Vector System 

**Figure 1:**
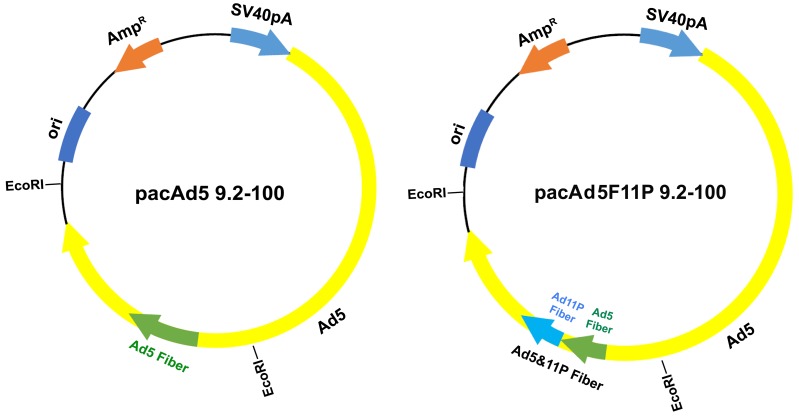
Modifications of the adenoviral backbone vector pacAd5 9.2-100. The proviral gene of units 9.2-100 of the *Ad5* and chimeric fiber Ad are diagramed. The chimeric *Ad5* and *Ad11P* fiber gene is indicated.

In order to generate a rAd vector that has high tropism to leukemia cells, we made a rAd vector that has a chimeric fiber of *Ad5* and *Ad11p* [18]. As the rAd backbone vector pacAd5 9.2-100 is too large to manipulate, we digested pacAd5 9.2-100 with EcoRI and ligated the Ad 8.5 Kb fragment, which contains the *Ad5* fiber gene, into pGEX-3Z plasmid, then mutated the *Ad5* fiber gene to be a chimeric gene of *Ad5* and *Ad11p*. Finally, we subcloned the Ad 8.5 Kb fragment back to the rAd backbone vector pacAd5 9.2-100, resulting in pacAd5F11P 9.2-100 (Figure 1). 

**Figure 2:**
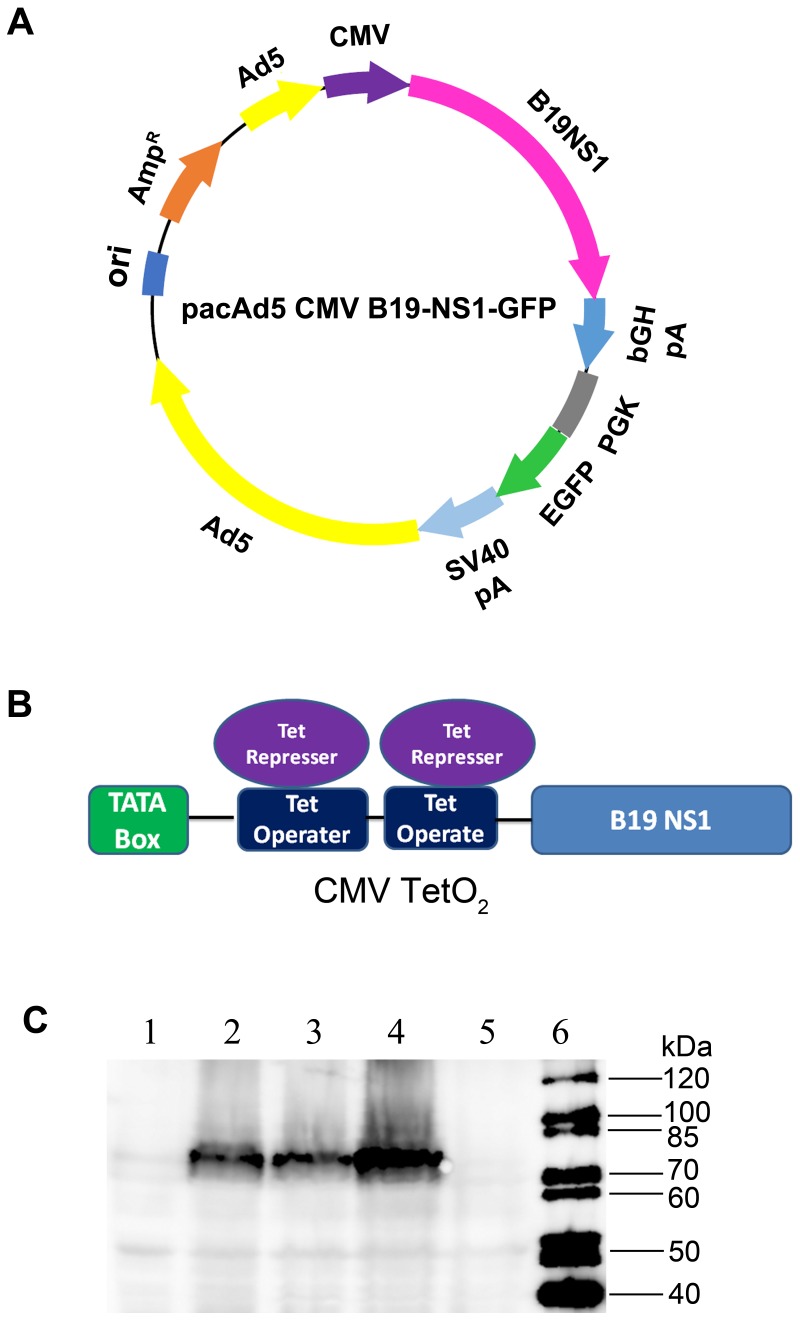
Modifications of the adenoviral shuttle vectors. (**A**,**B**) Modification of the CMV promoter. The transfer plasmid pacAd5 CMV-B19NS1-EGFP is diagrammed. Two tetracycline operator elements were inserted into the CMV promoter, which govern B19V NS1 expression. (**C**) Validation of the CMVTetO_2_ promoter activity. HEK293 cells and T-REx-293 cells were transfected with pacAd5 CMV-B19NS1-GFP and pacAd5 CMVTetO_2_-B19NS1-GFP, respectively. At 48 h post-transfection, cells were collected for Western blotting using anti-strep antibody to check B19V NS1 expression. Lane 1: HEK293 cells control; Lane 2: HEK293 cells transfected with pacAd5 CMV-B19NS1-GFP; Lane 3: HEK293 cells transfected with pacAd5 CMVTetO_2_-B19NS1-GFP; Lane 4: T-REx-293 cells transfected with pacAd5 CMV-B19NS1-GFP; Lane 5: T-REx-293 cells transfected with pacAd5 CMVTetO_2_-B19NS1-GFP; Lane 6: protein ladder.

It has been reported that autonomous parvoviruses, such as rat parvovirus H-1 (H-1PV), interfere with Ad replication due to the NS1 protein [22]. Therefore, we modified the CMV promoter in the shuttle plasmid, which transcribes B19V NS1-encoding mRNA, by inserting two tetracycline operator elements (Figure 2A,B). As a result, CMV promoter-driven NS1 expression was inhibited in the packaging cells, T-REx-293 cells, which constitutively expressed the tetracycline repressor [23], allowing a high-yield rAd production. As a control, the CMVTetO_2_ promoter still had activities in normal HEK293 cells (Figure 2C).

### 3.2. Production of B19V NS1-Expressing rAd

**Figure 3:**
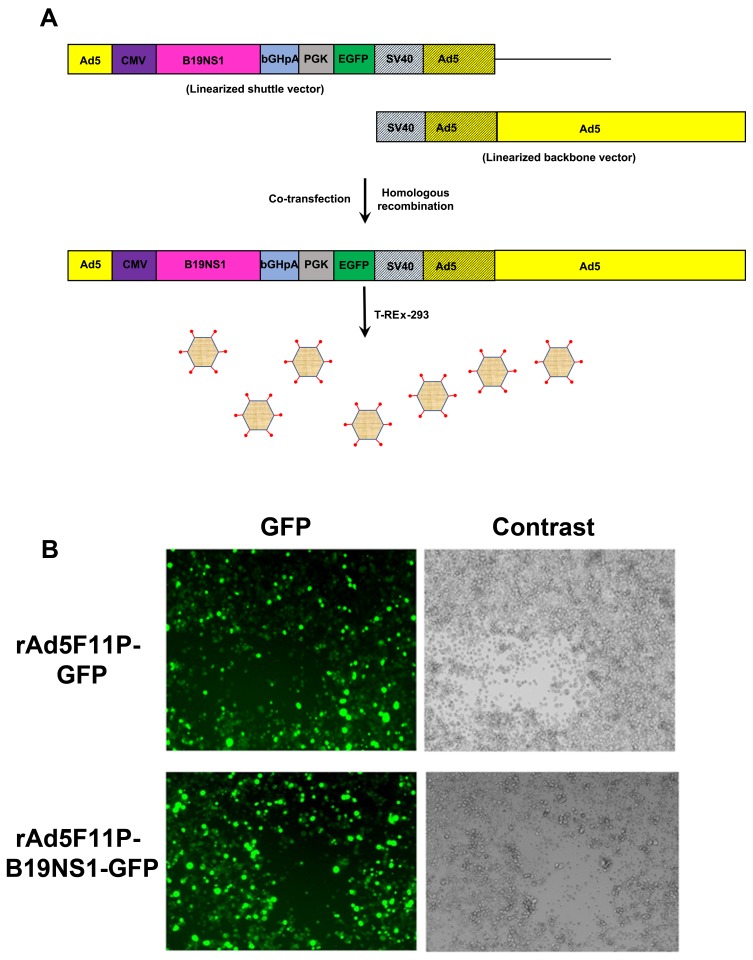
Production of recombinant adenovirus expressing GFP and B19NS1-GFP. (**A**) The schematic flowchart of recombinant Ad production. Both the Ad shuttle and proviral backbone plasmids are transfected into cells. After homologous recombination, a rAd proviral genome is generated, which further replicates to produce rAd virions as diagramed. (**B**) Recombinant adenovirus production. T-REx-293 cells were co-transfected using linearized pacAd5 CMVTetO_2_-GFP or pacAd5 CMVTetO_2_-B19NS1-GFP with linearized pacAd5F11P 9.2-100. After one week, GFP expression was monitored under a Nikon Eclipse Ti-S inverted microscope at 10× magnification.

T-REx-293 cells were co-transfected with linearized pacAd5 CMVTetO_2_-GFP or pacAd5 CMVTetO_2_-B19NS1-GFP and linearized pacAd5F11P 9.2-100 (Figure 3A). After one week, we found a high percentage (>80%) of GFP-expressing cells and a few plaques appeared, which were likely caused by rAd5F11P-GFP and rAd5F11P-B19NS1-GFP (Figure 3B). We then harvested the rAd-producing cells, released the viruses from cells, and saved the crude viral lysates (initial viral stocks). The initial viral stocks were used to amplify the virus in T-REx-293 cells, and the final viral lysates were then purified using CsCl gradient ultracentrifugation. The purified viruses were titrated by quantitative-PCR (qPCR) using a TaqMan probe targeting the *GFP* gene, which had titers of 5 × 10^12^ vgc/mL (viral genome copies (vgc) per mL). 

### 3.3. Ad5F11p-B19NS1-GFP Is More Effective in the Transduction of Leukemia Cells at a Relatively Low Multiplicity of Infection (MOI)

**Figure 4:**
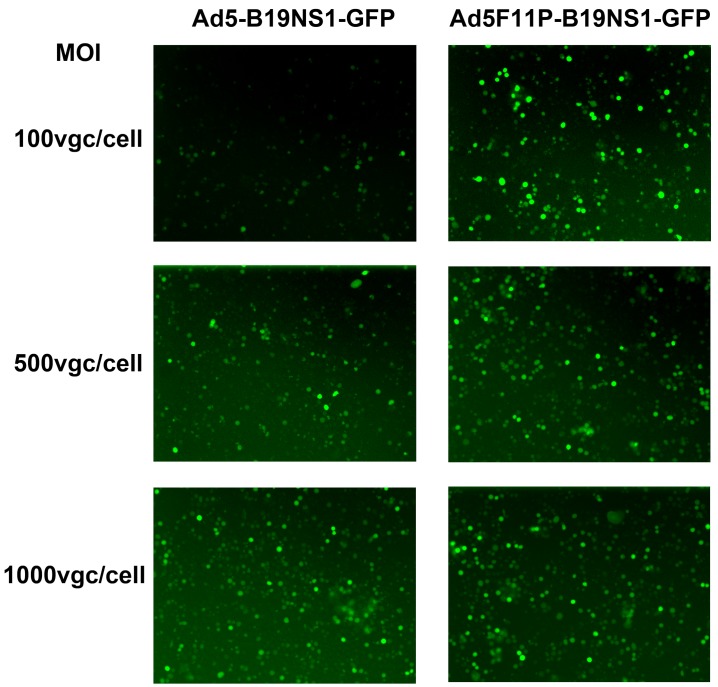
rAd5F11p-B19NS1-GFP is more effective than the parent rAd5 in the transduction of leukemia cells UT7/Epo-S1 at a relatively low MOI. UT7/Epo-S1 cells were transduced with rAd5-B19NS1-GFP or rAd5F11p-B19NS1-GFP at three different MOIs (100, 500, and 1000 vgc/cell), respectively. At 48 h post-transduction, GFP expression was monitored under a Nikon Eclipse Ti-S inverted microscope at 10× magnification.

We next transduced the UT7/Epo-S1 cells with rAd5F11p-B19NS1-GFP and rAd5-B19NS1-GFP, respectively, at various MOIs (viral genome copies (vgc) per cell). We found that at an MOI of 100, the rAd5F11p-B19NS1-GFP transduced >90% of UT7/Epo-S1 cells while rAd5-B19NS1-GFP transduced only <30%; at MOI of 500 and 1000, the transduction efficiency was over 90%, similar between transductions using rAd5F11p-B19NS1-GFP and rAd5-B19NS1-GFP (Figure 4). This result suggests that the rAd5F11p-B19NS1-GFP vector transduces UT7/Epo-S1 cells more efficiently than the rAd5-B19NS1-GFP vector, when they were used at a relatively low MOI. 

### 3.4. rAd5F11p-B19NS1-GFP Induces a Cell Cycle Arrest at G2 Phase and Apoptosis in Transduced Cells

**Figure 5:**
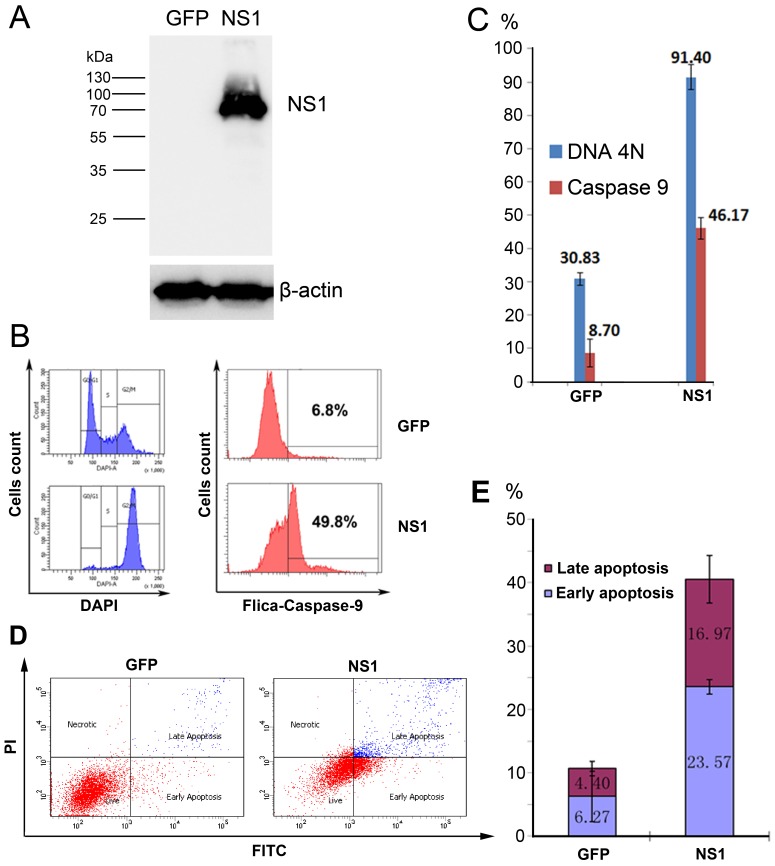
rAd5F11p-B19NS1-GFP induces cell cycle arrest at the G2/M phase and apoptosis in UT7/Epo-S1 cells. UT7/Epo-S1 cells were transduced with rAd5F11p-GFP or rAd5F11p-B19NS1-GFP at an MOI of 100 vgc/cell. After 48 h post-transduction, (**A**) Western blotting. Transduced cells were collected for Western blot analysis using an anti-strep antibody. The same membrane was reprobed by using anti-β-actin antibody. (**B**,**C**) Cell cycle analysis and Fluorescent-Labeled Inhibitors of Caspases (FLICA). Transduced cells were fixed and stained using 4′,6-diamidino-2-phenylindole (DAPI) or FLICA caspase-9. Cell cycle and caspase-9 activity were detected by flow cytometry. (**D**,**E**) Transduced cells were stained with Annexin-V FITC and Propidium Iodide (PI), followed by flow cytometry analysis. The percentages of both early and late apoptotic cells are presented with averages and standard deviations, which were obtained from at least three independent experiments.

In order to test our hypothesis to use B19V NS1 to kill leukemia cells, we transduced UT7/Epo-S1 cells with Ad5F11P-GFP (as a control) and Ad5F11p-B19NS1-GFP at an MOI of 100, respectively, to ensure over 90% of the cells were transduced. Western blotting was performed to detect the NS1 expression (Figure 5A). Cells were harvested at 48 h post-transduction and stained with DAPI to detect the cell cycle. We found that over 90% of the cells were arrested at G2/M (Figure 5B,C). This is consistent with our previous study that B19V NS1 protein is a key factor for disrupting the cell cycle at the G2/M phase [14].

Next, we looked into apoptosis induced by NS1 as previously reported [12]. Caspase-9 is an important member of the Caspase family and caspase-9 further processes other caspase members, including caspase-3 and caspase-7, to initiate a caspase cascade, which leads to apoptosis [24]. We chose caspase-9 activity as a marker to measure apoptosis. We found that compared with only 8.7% GFP-expressing cells, NS1 expression increased caspase-9 activity to 46.17% (Figure 5B,C). We also carried out Annexin V-FITC and PI double staining to confirm the NS1-induced apoptosis. The results showed, for GFP control, the apoptosis (early and late) percentage was 6.27% and 4.40%, respectively (a total 10.67%). For B19V NS1, the apoptosis (early and late) percentage was 23.57% and 16.97%, respectively (a total 40.54 %) (Figure 5D,E). 

Taken together, all of these results showed that chimeric fiber increased the tropism of the B19V NS1-expressing rAd to leukemia cells UT7/Epo-S1. The expressed NS1 induced over 90% of the leukemia cells arrested at the G2/M phase and the approximately 50% undergoing apoptosis. 

## 4. Discussion

In this study, we modified the *Ad5* fiber gene into a chimeric *Ad5* and *Ad11P* fiber gene to increase the transduction efficiency into leukemia cells UT7/Epo-S1. The fiber gene-modified rAd5F11P-B19NS1-GFP transduces UT7/Epo-S1 more effectively than the rAd5-B19NS1-GFP. More importantly, the transduction of NS1-expressing rAd vector induced nearly all the cells arrested at the G2/M phase. Cancer cells are considered as cells that cannot control their cell cycle progression. During tumorigenesis, due to genetic and epigenetic changes, the regulation of cell cycle is malfunctioned, resulting in uncontrolled cell proliferation [25]. Our novel rAd5F11P-B19NS1-GFP could be a promising gene-based therapeutic approach with anti-leukemia potential.

The cellular receptor for commonly used *Ad5* is coxsackie virus and adenovirus receptor (CAR) on the cell membrane. After the initial interaction between *Ad5* fiber with CAR, the second interaction between the RGD motif of the penton bases with α_v_β_3_ and α_v_β_5_ integrins facilitates *Ad5* internalization by endocytosis [26]. However, both CAR and α_v_β_3_ and α_v_β_5_ integrins are poorly expressed on the cell membrane of hematopoietic cells, thus rAd5 has a poor transduction efficiency into hematopoietic cells [5]. The interaction of the fiber knob with the cellular receptor is the key step by which Ad enters the cell. Different adenoviral serotypes exhibit different tissue tropism because their fiber proteins recognize and interact with different cellular receptors [27,28]. *Ad11p*, which belongs to adenovirus group B, has been reported to infect human CD34^+^ hematopoietic cells [6]. Thus, we replaced *Ad5* fiber with a chimeric fiber gene encoding the *Ad5* fiber tail domain and the *Ad11p* fiber shaft and knob domains. In our case, the chimeric *Ad5* and *Ad11P* fiber recognizes its cellular receptor on UT7/Epo-S1 cells more efficiently than the parent *Ad5* fiber, which could explain why the Ad5F11p-B19NS1-GFP is more effective in the transduction of UT7/Epo-S1 cells at a relatively low MOI than the rAd5-B19NS1-GFP. 

Our previous study has found that NS1 induces cell cycle arrest at the G2/M phase through its C-terminal transactivation domain, and NS1 transactivates signaling from ATR to CDC25C. Phosphorylated CDC25C prevents the dephosphorylation of CDK1 at tyrosine 15. Thus, an inactive cyclin B1–CDK1 complex is imported to the nucleus, thereby blocking progression from the G2- to the M-phase. We believe that the cell cycle arrest induced by NS1 in UT7/Epo-S1 is caused by the NS1-inactivated CDC25C. The CDC25C protein is a phosphatase responsible for the dephosphorylation and activation of CDK1 to promote the transition of cells to mitosis, so NS1-inactivated CDC25C could inhibit cancer cell proliferation. Apoptosis is a programmed cell death involving the sequential activation of caspases. The NS1-induced apoptosis activates caspase-9, indicating the intrinsic pathway, which is also called the mitochondrion-mediated pathway, which is different from the NS1-induced apoptosis during B19V infection [29]. 

In our study, we use modified rAd expressing B19V NS1 to induce cell cycle arrest and apoptosis in UT7/Epo-S1 cells at the G2/M phase, which shows a promising tool to kill the megakaryocytic leukemic cells. Our next step is to modify the promoter which controls NS1 expression into the hTERT promoter, which has activity specifically in cancer cells. Using this strategy, we could more specifically induce the cell cycle arrest and apoptosis of cancer cells. Thus, the rAd5F11P-B19NS1-GFP vector holds promise in gene-based therapeutics for anticancer potential to human leukemia.
